# Comparison of Chikungunya Virus and Zika Virus Replication and Transmission Dynamics in *Aedes aegypti* Mosquitoes

**DOI:** 10.4269/ajtmh.20-0143

**Published:** 2020-05-18

**Authors:** Alexis Robison, Michael C. Young, Alex D. Byas, Claudia Rückert, Gregory D. Ebel

**Affiliations:** 1 Department of Microbiology, Immunology and Pathology, College of Veterinary Medicine and Biomedical Sciences, Colorado State University, Fort Collins, Colorado;; 2 Department of Biochemistry and Molecular Biology, College of Agriculture, Biotechnology and Natural Resources, University of Nevada, Reno, Nevada

## Abstract

Chikungunya virus (CHIKV) and Zika virus (ZIKV) are arthropod-borne viruses transmitted mainly by *Aedes aegypti* mosquitoes. These viruses have become endemic in large parts of North, Central, and South America. Arboviruses persistently infect mosquitoes throughout their life span and become infectious (i.e., expectorate infectious virus in saliva) after a period of time called the extrinsic incubation period (EIP). The duration of this infectiousness, however, is not well characterized. This is an important shortcoming because many epidemiological models assume that mosquitoes continue to be infectious for the duration of their life span. To define the duration of infectiousness for CHIKV and ZIKV, mosquitoes were infected orally with these viruses. Every 2 days, legs/wings, midguts, salivary glands, and saliva were collected from 30 to 60 mosquitoes and viral load measured. In CHIKV-infected mosquitoes, infectious virus in saliva peaked early (2–4 dpi), and then decreased rapidly and was rarely observed after 10 dpi. Viral RNA in infected tissues also decreased after the initial peak (4–8 dpi) but did so much less drastically. In ZIKV-infected mosquitoes, the infectious virus in saliva peaked at 12–14 dpi and dropped off only slightly after 14 dpi. In infected tissues, viral RNA increased early during infection, and then plateaued after 6–10 days. Our findings suggest that significant variation exists in the duration of the infectious period for arboviruses that is in part influenced by virus clearance from expectorated saliva.

## INTRODUCTION

Chikungunya virus (CHIKV; genus *Alphavirus*) and Zika virus (ZIKV; genus *Flavivirus*) are arthropod-borne viruses transmitted by *Aedes aegypti* and *Aedes albopictus* mosquitoes. Both viruses have spread across the world over the last two decades and have recently become endemic in large parts of North, Central, and South America.^1–3^ Clinical symptoms of Chikungunya fever and Zika fever are very similar: fever, arthralgia, myalgia, rash, and conjunctivitis.^4^ However, both viruses can cause more serious diseases including persistent arthralgia (CHIKV) or neurological symptoms (CHIKV and ZIKV), although neurological complications are extremely rare in CHIKV infections.^5^ Zika virus has also been known to cause Zika congenital syndrome,^6^ which is a collective term for neurodevelopmental disorders in infants as a result of ZIKV infection of the developing fetus in pregnant women.

Arboviruses persistently infect mosquitoes throughout their life span of approximately 2–4 weeks in nature,^7^ but it is unknown whether virus titers remain consistent in all tissues over time. When competent mosquito vectors take a blood meal containing virus, the virus first replicates in the midgut, then disseminates through the mosquito body, and finally reaches the salivary glands, where it may be transmitted with saliva during a subsequent blood meal.^8^ The period between blood meal and transmission capability is called the extrinsic incubation period (EIP). The duration of the EIP is a complex phenotype that is determined by specific virus–mosquito pairings and influenced strongly by environmental conditions.^9–12^ Formulas to quantitatively estimate the basic reproductive rate (*R*_0_) for a vector-borne pathogen (e.g., vectorial capacity)^13^ include only a term for the duration of the EIP but not for loss of transmissibility. Therefore, epidemiological models tend to implicitly assume that mosquitoes are infectious for the duration of their life span.

During a previous study, we observed that the proportion of CHIKV RNA-positive saliva was higher at an earlier time point than a later time point.^14^ This was not observed in mosquitoes infected with ZIKV. However, we did not evaluate infectious virus present in the saliva, and only two time points were taken in the study.^14^ We therefore sought to determine whether virus replication and transmission dynamics differ between these two viruses. We thus investigated the dynamics of replication, dissemination, and estimated transmission of CHIKV and ZIKV in a Mexican colony of *Ae. aegypti* mosquitoes to see when virus in the saliva peaks and whether transmission drops off over time. We exposed *Ae. aegypti* mosquitoes to a blood meal containing CHIKV or ZIKV, sorted blood-fed mosquitoes into individual tubes, and then collected the midguts, legs/wings, salivary glands, and saliva every 2 days from 2 to 20 days post-infection (dpi). We used infectious virus in collected saliva as a proxy for transmission efficiency. Transmission experiments using live animals may provide results that differ from ours, but saliva collection is the most feasible method to estimate transmission in vector competence studies. We found that in mosquitoes infected with CHIKV, viral RNA copies and amount of infectious virus in saliva peaked early and gradually decreased over time. In ZIKV-infected mosquitoes, viral RNA and infectious virus peaked later and persisted in saliva throughout the experiment. Overall, our study provides evidence that virus transmission may not always be maintained throughout the mosquito’s life span, and that the rate of virus clearance from saliva varies in a virus–mosquito–specific manner. This finding has clear implications for modeling the epidemiology and transmission of arboviruses.

## MATERIALS AND METHODS

### Mosquitoes.

*Aedes aegypti* mosquitoes from Poza Rica, Mexico, were colonized in 2012.^15^ Mosquito husbandry and insectary practices are as described elsewhere.^3^ Briefly, larvae were raised on a diet of powdered fish food, and adult mosquitoes were maintained at 28°C with a 12:12 (L:D) photoperiod and 70–80% relative humidity. Water and sugar were provided ad libitum.

### Cells and viruses.

Vero cells (ATCC^®^ CCL-81™, Manassas, VA) were maintained in Dulbecco’s modified Eagle medium (DMEM) supplemented with 5% fetal bovine serum (FBS), 50 µg/mL gentamycin, and 100 µg/mL penicillin/streptomycin at 37°C in a 5% CO_2_-containing humidified atmosphere.

Chikungunya virus 99659 (British Virgin Islands strain; GenBank: KJ451624.1; Vero passage 2) and ZIKV PRVABC59 (Puerto Rican strain; GenBank: KU501215.1; Vero passage 4) were propagated in Vero cells, and single use aliquots were prepared and frozen at −80°C. Stocks were titrated on Vero cells by the standard plaque assay. Briefly, cells were infected at ∼90% confluency using 10-fold serial dilutions of virus in DMEM (without FBS). After 1 hour of incubation, an overlay was added, consisting of 6 g/L Tragacanth gum (MP Biomedicals Cat #104792, Irvine, CA) in Eagle’s Minimum Essential Medium (EMEM) supplemented with 4% FBS, 100 µg/mL gentamycin, 100 µg/mL penicillin/streptomycin, and 5 µg/mL amphotericin B. The overlay was removed 3 dpi (CHIKV) or 5 dpi (ZIKV), and cells were fixed in 20% ethanol containing 1 g/L crystal violet. Stock titers were 1.8 × 10^7^ PFU/mL and 3 × 10^7^ plaque forming units (PFU)/mL for CHIKV and ZIKV, respectively.

### Virus infection of mosquitoes and sample collection.

Female mosquitoes, 5–7 days old, were fed an infectious blood meal of defibrinated calf blood containing 9 × 10^6^ PFU/mL of CHIKV or 1.5 × 10^7^ PFU/mL of ZIKV in 20% DMEM (1:1 mix of blood:virus stock). The artificial blood meal contents were added to water-jacketed glass feeders sealed with hogs gut and connected to a 37°C water bath. Mosquitoes were allowed to feed for approximately 45 minutes, after which they were anesthetized at 4°C and sorted. Engorged female mosquitoes were transferred to individual 50-mL conical tubes with a small piece of paper towel at the bottom. The tops of the conical tubes were covered in mesh and secured with a rubber band. After females began to recover, water was added directly to the conical tube until a small pool collected at the bottom after absorption into the paper towel. Two filter cards were then placed on top of each tube as a sugar source for the duration of the experiment. One card was soaked in 20% sucrose solution, whereas the other was soaked in a solution of 50% honey and 20% sucrose.

Mosquitoes were then held for up to 20 days in a BSL-3/ACL-3 insectary under the same conditions as described earlier. Every 2 days following the infectious blood meal, 30 mosquitoes were cold-anesthetized. Legs/wings were collected first, and then saliva was collected by inserting the mosquito proboscis into a capillary tube containing immersion oil for 30 minutes. After salivation, the midguts and salivary glands were dissected. Legs/wings, midguts, and salivary glands were placed directly into lysis buffer (Thermo Fisher Scientific, MagMax total RNA isolation kit). Capillary tubes containing saliva were placed in microcentrifuge tubes with 100 µL mosquito diluent (1 × PBS containing 20% FBS, 50 µg/mL penicillin/streptomycin, 50 µg/mL gentamycin, and 2.5 µg/mL Fungizone) and centrifuged at 15,000 × *g* for 5 minutes at 4°C to expel saliva-containing oil into mosquito diluent before freezing. All samples were stored at −80°C until sample processing. Experiments were performed twice for CHIKV and ZIKV infections.

### Virus titration of saliva samples.

Saliva samples were titrated as previously described^16^ with minor modifications. Vero cells were seeded in 12-well plates and allowed to reach ∼90% confluency. Saliva samples were thawed, briefly vortexed, and centrifuged for 3 minutes at 15,000 × *g*. In the meantime, Vero cell culture media was removed and replaced with 200 µL of DMEM without additives. Then, 30 µL of saliva sample were added to each well. Plates were rocked for 10 minutes at room temperature to allow spread of virus over the monolayer, returned to the 37°C CO_2_ incubator, and briefly rocked every 15 minutes for 1 hour. After 1 hour, 1 mL of Tragacanth/media overlay was added to each well, and plates were incubated at 37°C. After 3 days (for CHIKV) or 5 days (for ZIKV), the plates were fixed with a staining solution (1 g/L crystal violet in 20% ethanol solution). Plaques were visualized on a light box, counted, and recorded. For samples with high viral titers, this process was repeated with 10-fold dilutions of input sample to permit accurate plaque quantification. The detection limit for plaque assays was 3.33 PFU/saliva sample.

### RNA extraction.

Legs/wings, midgut, and salivary gland samples were thawed, vortexed thoroughly, and briefly centrifuged. Viral RNA was extracted from individual samples using the MagMax total RNA isolation kit (Thermo Fisher Scientific Cat #AM1830), following the manufacturer’s protocol, and the KingFisher Flex System. Samples were eluted in 50 µL of nuclease-free water, of which 5 µL were placed directly into quantitative real-time reverse transcriptase–PCR (qRT-PCR), and the rest was collected into 8-strip tubes and stored at −80°C.

### Quantitative real-time reverse transcriptase–PCR.

One-step qRT-PCR was performed on individual samples of legs/wings, midguts, salivary glands, and saliva with specific probes and primers for CHIKV and ZIKV in a multiplex reaction, as previously described.^3^ Positive RNA standards were generated for both viruses^3^ and included in each qRT-PCR plate, as well as negative controls. Saliva samples were thawed, vortexed, and briefly centrifuged, and 5 µL were used directly for qRT-PCR. The limit of reliable detection for qRT-PCR was 335 viral copies/sample.

## RESULTS

### Chikungunya virus replication and transmission dynamics in *Ae. aegypti* mosquitoes.

**Figure 1.**
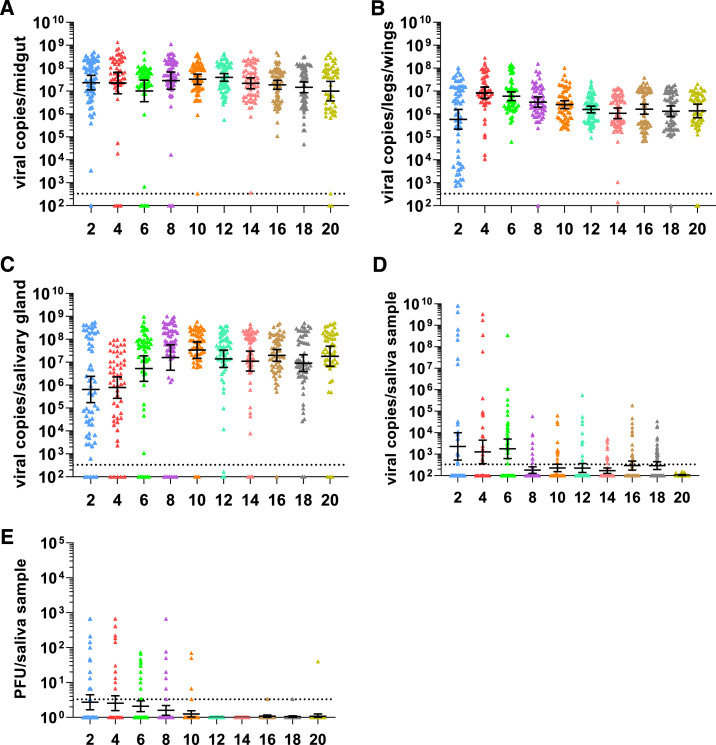
Chikungunya virus (CHIKV) RNA copy numbers and infectious virus in *Aedes aegypti* mosquito tissues. Viral RNA copy numbers in CHIKV-infected midguts (**A**), legs/wings (**B**), salivary glands (**C**), and saliva (**D**) from 2 to 20 dpi are shown. All viral copy numbers were quantified using quantitative real-time reverse transcriptase–PCR. Infectious virus titers in saliva samples (**E**) from 2 to 20 dpi were titrated by plaque assay on Vero cells. The geometric mean of 50–60 mosquito samples from two replicate experiments is shown. Error bars indicate the 95% CI. The limit of detection was 335 copy numbers (correlates to 36.5 CT value) for viral RNA and 3.3 PFU for infectious virus. This figure appears in color at www.ajtmh.org.

We were initially interested in whether infectious CHIKV detected in the saliva of *Ae. aegypti* mosquitoes continues to be present throughout the mosquito’s lifetime (i.e., estimate of transmission dynamics). Chikungunya virus infection rates were high (94–100%) for all time points, as measured by viral RNA in the midgut (Table 1). Dissemination of the virus occurred rapidly throughout the mosquito, with all of the legs/wings and most of the salivary glands being infected within 2 dpi (Table 1). Viral RNA was detected in the saliva of a subset of mosquitoes from 2 dpi up to 18 dpi, with the highest percentage of RNA-positive saliva (46.7%) at 6 dpi (Table 1). Infectious virus was also detected in saliva by 2 dpi but declined rapidly and was not detected after 10 dpi, with the exception of one saliva sample at 20 dpi (Table 1). Viral RNA levels peaked at 4 dpi in midguts (Figure 1A), at 4 dpi in legs/wings (Figure 1B), at 8 dpi in salivary glands (Figure 1C), and 6 dpi in saliva (Figure 1D). Viral RNA then gradually decreased from peak to 20 dpi in midguts (2.7-fold), legs/wings (6-fold), and salivary glands (1.9-fold) (Figure 1A–C). In the saliva, viral RNA dropped more drastically than in the other tissues (Figure 1D and E). Infectious virus titers peaked at 2 dpi, with a mean titer of 165 PFU per virus-positive saliva sample, and titers remained consistent until they dropped off completely at 12 dpi (Figure 1E).

**Table 1:** Number of Chikungunya virus (CHIKV)–positive midguts, legs/wings, salivary glands, and saliva in *Aedes aegypti* mosquitoes

Dpi	2	4	6	8	10	12	14	16	18	20
Midguts	59/60 (98%)	60/60 (100%)	60/60 (100%)	60/60 (100%)	60/60 (100%)	60/60 (100%)	60/60 (100%)	60/60 (100%)	60/60 (100%)	47/50 (94%)
Legs/wings	60/60 (100%)	60/60 (100%)	60/60 (100%)	59/60 (98%)	60/60 (100%)	60/60 (100%)	59/60 (98%)	60/60 (100%)	59/60 (98%)	48/50 (96%)
Salivary glands	55/60 (92%)	55/60 (92%)	56/60 (93%)	56/60 (93%)	57/60 (95%)	58/60 (97%)	56/60 (93%)	49/60 (82%)	58/60 (97%)	47/50 (94%)
Saliva (RNA)	19/60 (32%)	19/60 (32%)	28/60 (47%)	6/60 (10%)	4/60 (7%)	8/60 (13%)	5/60 (8%)	8/60 (13%)	11/60 (18%)	0/50 (0%)
Saliva (PA)	17/60 (28%)	16/60 (27%)	11/60 (18%)	6/60 (10%)	3/60 (5%)	0/60 (0%)	0/60 (0%)	0/60 (0%)	0/60 (0%)	0/50 (0%)

**Figure 2.**
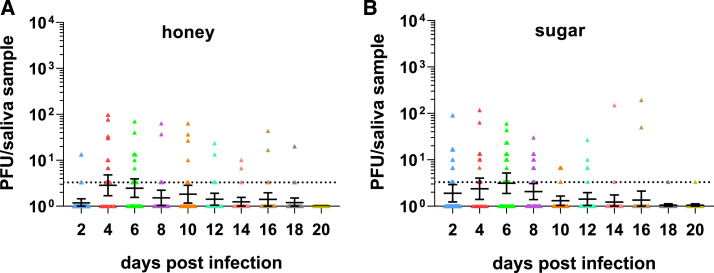
Chikungunya virus (CHIKV) infectious virus in saliva of *Aedes aegypti* mosquitoes fed on honey or sugar. Chikungunya virus was titrated in saliva samples of *Ae. aegypti* mosquitoes fed on either a filter card soaked in honey/sugar solution (**A**) or a sugar cube (**B**) following infection. Mosquito infection status was confirmed in mosquito leg and body tissues by plaque assay. The geometric mean and individual data points from 30 mosquito samples are shown. Error bars indicate the 95% CI. The limit of detection was 3.3 PFU per saliva sample. This figure appears in color at www.ajtmh.org.

We also assessed the impact of sugar type on measurement of infectious virus in saliva to eliminate the possibility that honey has an impact on CHIKV transmission. We thus compared CHIKV infectious virus in saliva of mosquitoes fed on honey-soaked filter cards with virus in saliva of mosquitoes fed on a sugar cube. The proportion of infected saliva samples and the viral loads in these samples were similar (Figure 2).

### Zika virus replication and transmission dynamics in *Ae. aegypti* mosquitoes.

**Figure 3.**
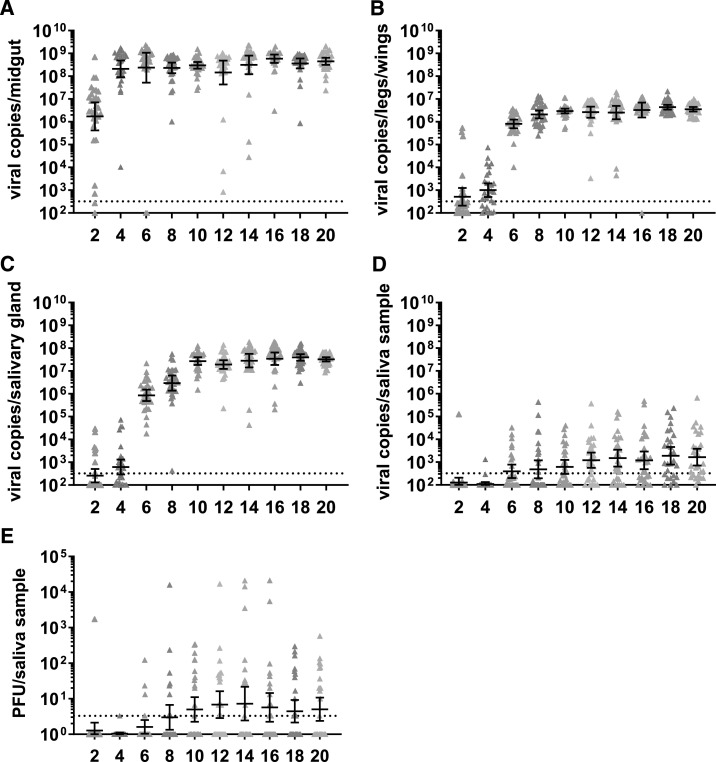
Zika virus (ZIKV) RNA copy numbers and infectious virus in *Aedes aegypti* mosquito tissues. Viral RNA copy numbers in ZIKV-infected midguts (**A**), legs/wings (**B**), salivary glands (**C**), and saliva (**D**) from 2 to 20 dpi are shown. All viral copy numbers were quantified using quantitative real-time reverse transcriptase–PCR. Infectious virus titers in saliva samples (**E**) from 2 to 20 dpi were titrated by plaque assay on Vero cells. The geometric mean of 30 mosquito samples from one experiment is shown. Error bars indicate the 95% CI. The limit of detection was 335 copy numbers (correlates to 36.5 CT value) for viral RNA and 3.3 PFU for infectious virus. This figure appears in color at www.ajtmh.org.

Based on our observations in the CHIKV-infected mosquitoes, we wanted to see whether the trends were the same for replication and estimated transmission dynamics in ZIKV-infected *Ae. aegypti* mosquitoes. Zika virus infection was also high (93.3–100%), as measured by viral RNA in the midgut (Table 2). Dissemination of the virus was slower than CHIKV, with all of the salivary glands being infected by 6 dpi (Table 2). Viral RNA was detected in the saliva of a subset of mosquitoes from 2 dpi up to 20 dpi, with the highest percentage of RNA-positive saliva (63.3%) at 18 dpi (Table 2). Infectious virus was also detected in saliva as early as 2 dpi and continued to be present throughout the experiment with the exception of 4 dpi (Table 2). At the peak of virus transmission (12–14 dpi), 46.7% of mosquitoes contained infectious virus in their saliva. Zika virus RNA reached peak levels at 6 dpi in midguts (Figure 3A), at 10 dpi in legs/wings (Figure 3B), at 10 dpi in salivary glands (Figure 3C), and at 12 dpi in saliva (Figure 3D). Viral RNA load was consistent throughout the experiment in midguts, legs/wings, and salivary glands (Figure 3A–C). In saliva, viral RNA increased over time and remained consistent up to 20 dpi (Figure 3D). Infectious virus titers peaked at 14 dpi, with a mean titer of 2800 PFU per virus-positive saliva sample. Titers dropped off only slightly afterward (Figure 3E).

**Table 2:** Number of Zika virus–positive midguts, legs/wings, salivary glands, and saliva in *Aedes aegypti* mosquitoes

Dpi	2	4	6	8	10	12	14	16	18	20
Midguts	29/30 (97%)	30/30 (100%)	28/30 (93%)	30/30 (100%)	30/30 (100%)	30/30 (100%)	30/30 (100%)	30/30 (100%)	30/30 (100%)	30/30 (100%)
Legs/wings	21/30 (70%)	26/30 (87%)	30/30 (100%)	30/30 (100%)	30/30 (100%)	30/30 (100%)	30/30 (100%)	29/30 (97%)	30/30 (100%)	30/30 (100%)
Salivary glands	13/30 (43%)	25/30 (83%)	30/30 (100%)	30/30 (100%)	29/30 (97%)	30/30 (100%)	29/30 (97%)	29/30 (97%)	30/30 (100%)	30/30 (100%)
Saliva (RNA)	1/30 (3%)	1/30 (3%)	10/30 (33%)	10/30 (33%)	12/30 (40%)	18/30 (60%)	16/30 (53%)	16/30 (53%)	19/30 (63%)	16/30 (53%)
Saliva (PA)	1/30 (3%)	0/30 (0%)	4/30 (13%)	6/30 (20%)	11/30 (37%)	14/30 (47%)	14/30 (47%)	12/30 (40%)	10/30 (33%)	12/30 (40%)

### Comparison of CHIKV and ZIKV in mosquito saliva.

**Figure 4.**
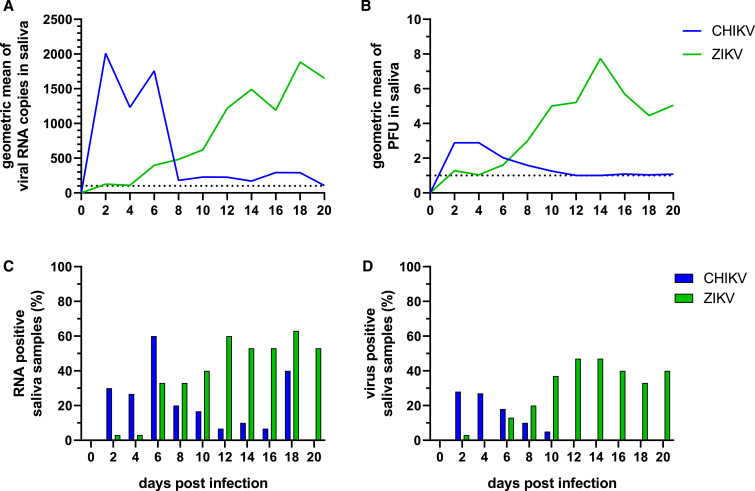
General trends of Chikungunya virus (CHIKV) and Zika virus (ZIKV) RNA copy numbers and infectious virus in *Aedes aegypti* mosquito saliva. Geometric mean of viral RNA copy numbers (**A**) and infectious virus (**B**) from CHIKV and ZIKV saliva samples from 2 to 20 dpi are shown. Percent of positive samples of viral RNA copy numbers (**C**) and infectious virus (**D**) from CHIKV and ZIKV saliva samples from 2 to 20 dpi are shown. This figure appears in color at www.ajtmh.org.

We calculated the geometric mean titer for all saliva samples on each sampling day to compare transmission dynamics of CHIKV and ZIKV (Figure 4A and B). We also used the percentage of positive samples on each day from Tables 1 and 2 to compare the transmission rates and infectious virus levels between CHIKV and ZIKV (Figure 4C and D). Overall, CHIKV RNA and infectious virus decreased in saliva after 4–6 dpi, whereas ZIKV RNA and infectious virus increased throughout the period of observation (Figure 4A and B). We observed the same trend for the percent of positive saliva samples (Figure 4C and D).

## DISCUSSION

Here, we compared CHIKV and ZIKV replication and transmission in a colony of *Ae. aegypti* mosquitoes from Poza Rica, Mexico, to explore the hypothesis that the duration of infectiousness of CHIKV and ZIKV may differ. We estimated virus transmission by measuring infectious virus in mosquito saliva and found that although CHIKV transmission peaked early (2 dpi) and declined thereafter, ZIKV transmission peaked later (14 dpi) and remained more consistent over time. Chikungunya virus RNA levels in saliva also decreased after 6 dpi, but numerous mosquito saliva samples remained CHIKV RNA positive up to 18 dpi. This highlights the importance to measure infectious virus in vector competence studies.

We had observed a similar, albeit less dramatic, decrease in CHIKV RNA in saliva between time points in a previous study.^14^ However, although the same virus isolate and mosquito colony were used, the experimental setup was quite different. Fewer time points were used, infectious virus was not determined, and female mosquitoes were sequentially blood-fed in various chronological orders with uninfected blood, blood containing ZIKV or blood containing CHIKV. In the latter study,^14^ all mosquitoes tested had thus received at least two blood meals, which may explain overall higher numbers of viral RNA-positive saliva samples of these mosquitoes.^17^ It has recently been shown that a second noninfectious blood meal (3 days after the initial infectious blood meal) can enhance CHIKV dissemination and transmission.^17^ It may thus be possible that a second blood meal could have resulted in renewed or prolonged transmission of CHIKV at later times after infection. *Aedes aegypti* mosquitoes tend to take multiple smaller blood meals (“sipping” behavior), due to interruptions in feeding and necessary resources for oviposition.^18^ Although multiple smaller blood meals thus represent what happens in nature, using one infectious blood meal for vector competence experiments is common practice and makes our data relevant to researchers selecting time points to determine vector competence for CHIKV. Many studies evaluate transmission at late time points, such as 14 dpi, which may not reflect peak transmission for CHIKV.

We were surprised to observe lower overall transmission levels for CHIKV than some previous studies using this virus strain and the same mosquito colony.^3,14,19^ Both Rückert et al.^3^ and Magalhaes et al.^14^ focused on the study of viral coinfections and, thus, measured only viral RNA in saliva because of the inherent difficulty of plaque-titrating two viruses from the same sample. The estimated transmission rates thus cannot be directly compared. In Sanchez-Vargas et al.^19^ study, the authors investigated virus titers from *Ae. aegypti* and *Ae. albopictus* saliva and salivary glands infected with CHIKV. The authors detected infectious virus in 50% of saliva samples at 9 dpi for this particular virus–mosquito combination (*Ae. aegypti* from Poza Rica and CHIKV strain 99659), which is significantly higher than our estimated transmission rate of 10% at 8 dpi. However, saliva sampling and virus titration methods also varied slightly between our study and Sanchez-Vargas et al.^19^ study, and infectious virus titers of saliva samples 9 dpi were also low in the latter study (76.5 mean PFU/mL, corresponding to ∼23 PFU/saliva sample), despite a higher proportion of positive saliva samples.

In conclusion, the results from our study suggest that once a mosquito is infected with CHIKV and able to transmit virus, it may not necessarily continue to do so for the remainder of its life. This contrasts to the general assumption that the EIP of a mosquito presents an end point after which the mosquito will transmit virus with each subsequent blood meal. Our results could impact further considerations for the EIP of CHIKV-infected mosquitoes and how vectorial capacity is calculated, and they may provide insight into saliva sampling for vector competence experiments. The observed discrepancy between CHIK RNA and infectious virus in mosquito saliva also further highlights the importance of sampling infectious virus whenever possible because RNA copies may not correlate directly to infectious virus.
